# Testosterone therapy-induced erythrocytosis: can phlebotomy be justified?

**DOI:** 10.1530/EC-24-0283

**Published:** 2024-09-28

**Authors:** Peter Bond, Tijs Verdegaal, Diederik L Smit

**Affiliations:** 1 Department of Internal Medicine, Elisabeth TweeSteden Hospital, Tilburg, the Netherlands; 2 Department of Performance and Image-enhancing Drugs Research, Android Health Clinic, Utrecht, the Netherlands; 3 Department of Internal Medicine, Spaarne Gasthuis, Haarlem, the Netherlands

**Keywords:** androgens, erythrocytosis, phlebotomy, polycythemia, testosterone

## Abstract

Erythrocytosis, or elevated hematocrit, is a common side effect of testosterone therapy (TTh) in male hypogonadism. Testosterone stimulates erythropoiesis through an initial rise in erythropoietin (EPO), the establishment of a new EPO/hemoglobin ‘set point’, and a parallel decrease in the master iron regulator protein hepcidin, as well as several other potential mechanisms. Evidence shows an increased thrombotic risk associated with TTh-induced erythrocytosis. Several guidelines by endocrine organizations for the treatment of male hypogonadism recommend against starting TTh in patients presenting with elevated hematocrit at baseline or stopping TTh when its levels cannot be controlled. Besides dose adjustments, therapeutic phlebotomy or venesection is mentioned as a means of reducing hematocrit in these patients. However, evidence supporting the efficacy or safety of therapeutic phlebotomy in lowering hematocrit in TTh-induced erythrocytosis is lacking. In light of this dearth of evidence, the recommendation to lower hematocrit using therapeutic phlebotomy is notable, as phlebotomy lowers tissue oxygen partial pressure (pO_2_) and eventually depletes iron stores, thereby triggering various biological pathways which might increase thrombotic risk. The potential pros and cons should therefore be carefully weighed against each other, and shared decision-making is recommended for initiating therapeutic phlebotomy as a treatment in patients on TTh who present with increased hematocrit.

## Introduction

Testosterone therapy (TTh) is prescribed to hypogonadal men to correct testosterone levels and alleviate symptoms that result from its deficiency. Benefits include improved sexual function and quality of life (1), improvements in body composition and muscle strength (2), increased bone mineral density (3), and correction of anemia (4). However, as with any medical therapy, its benefits need to be carefully weighed against its potential side effects. Notably, whereas its stimulating effect on erythropoiesis can be beneficial for the correction of anemia in some patients, the same effect can lead to erythrocytosis in others* –* with a nearly fourfold greater risk for erythrocytosis compared with placebo (5). Erythrocytosis, sometimes also called polycythemia, is defined as a red cell mass (RCM) >25% above the mean normal predicted value (6). Since measuring RCM is expensive, cumbersome, and virtually unavailable for the clinician, hematocrit or hemoglobin levels are used as a surrogate.

There is concern that elevated hematocrit carries an increased risk for thrombotic events. Several guidelines therefore mention hematocrit cutoff values: either as a contraindication to start TTh or to stop TTh (7, 8, 9, 10, 11, 12). This concern historically stems from erythrocytosis induced by myeloproliferative disorders, such as polycythemia vera (PV), of which increased red cell mass or hematocrit is one of the hallmarks and major criteria in diagnosis, and thrombosis is the major cause of morbidity and mortality (6). Consequently, therapeutic phlebotomy with a hematocrit target <45% is the mainstay in the treatment of PV to prevent thrombosis (6). Indeed, several guidelines for the treatment of male hypogonadism also discuss phlebotomy as a means of reducing hematocrit in TTh-induced erythrocytosis (7, 8, 9, 10, 11) (Table 1), although some, such as the UK Society for Endocrinology (12), do not.

**Table 1:** Testosterone therapy (TTh) guideline statements or recommendations from several endocrine organizations regarding phlebotomy or venesection for TTh-induced hematocrit elevations.

Organization	Statement regarding phlebotomy/venesection
Endocrine Society (7)	*Using therapeutic phlebotomy to lower hematocrit is also effective in managing T treatment–induced erythrocytosis.*
Endocrine Society of Australia (8)	*It (elevated hematocrit) can usually be managed by reduction of the testosterone dose (and/or frequency), but may rarely require venesection.*
European Academy of Andrology (9)	*There is a general consensus that hematocrit >54% requires TRT withdrawal (and sometimes phlebotomy) to minimize the risks of VTE and CV events.*
British Society for Sexual Medicine (10)	*Haematocrit levels should remain below 54% (LoE 2, Grade B). Dose adjustments and/or periodic venesection may be required in order to achieve this.*
European Association of Urology (11)	*An elevated haematocrit in the absence of any comorbidities, or acute CV or venous thromboembolism, can be managed by a reduction in testosterone dose – a change in formulation; conversely, if the elevated haematocrit is very high, it can be managed by venesection (500 mL), even repeated if necessary, with usually no need to stop the testosterone therapy.*

However, there are important differences between PV (and other causes of primary erythrocytosis) and secondary erythrocytosis, with good arguments against hematocrit being a driver of thrombotic risk (13). As such, the efficacy and safety of an intervention such as phlebotomy to decrease hematocrit should therefore be critically assessed per subtype of erythrocytosis. A testosterone dosage reduction with one or two phlebotomies to accelerate the correction of hematocrit* –* with the dose reduction in order to persist this* –* is unlikely to cause harm. This, however, might be different for periodic phlebotomy in order to maintain decreased (or ‘corrected’) hematocrit in case a dose reduction is insufficient or compromises symptomatic relief from TTh. After all, periodic phlebotomy does more than merely decrease hematocrit. The persistent suppression of hematocrit follows after depletion of iron stores* –* as it puts the proverbial brakes on erythropoiesis by lack of substrate* –* and lowers tissue oxygen partial pressure (pO_2_), thereby triggering various biological pathways. Some of these pathways might affect thrombotic risk (14).

In this article, we will describe how TTh is thought to induce erythropoiesis, discuss the evidence of TTh-induced erythrocytosis as a cause of thrombosis, and conclude on the potential beneficial or detrimental impact of providing therapeutic phlebotomy as a ‘treatment’ for it.

## Testosterone therapy-induced erythrocytosis

The stimulatory effect of TTh on erythropoiesis in male hypogonadism was already recognized in the 1940s (15). Its use as a treatment for certain forms of anemia gained traction in the 1970s and remained in use until the dawn of recombinant erythropoietin (EPO) in the late 1980s (16). Nevertheless, administration of androgens still remains in use for the treatment of anemia to some extent today. The stimulatory effect of testosterone on erythropoiesis is dose-dependent, more pronounced in older compared with younger men (17), and is unlikely to depend on its conversion to the more potent androgenic metabolite 5α-dihydrotestosterone (DHT) by the 5α-reductase family of enzymes (18, 19). Short-acting parenteral testosterone depots are thought to be associated with a greater risk of erythrocytosis compared with other formulations (20). This is likely caused by the transient supraphysiological peak testosterone concentrations that these depots more easily cause than other formulations (21). However, head-to-head trials are largely lacking and a recent network meta-analysis of randomized-controlled trials concluded that all TTh formulations are associated with an increase in hematocrit with only intramuscular testosterone enanthate/cypionate being more potent in doing so compared with transdermal testosterone patches, but with no differences between other treatment modalities (22). No TTh formulation exceeded a pooled mean increase of 4.3%pt. Importantly, differences in dosing, i.e., target testosterone levels, might obfuscate differences between formulations.

In large randomized-controlled trials, the number of participants exceeding hematocrit thresholds varies. In the TRAVERSE trial* –* the largest randomized-controlled trial of TTh to date comprising 5246 men* –* six participants (<1%) exceeded the hematocrit threshold of 54% even after titration to the lowest dosage (20.25 mg testosterone gel daily), requiring them to discontinue treatment (23). In contrast, in the T4DM trial, hematocrit increased to 54% or higher in 106 (22%) of participants randomized to TTh, with 25 participants (5%) discontinuing as a result (24). Important differences between these two landmark trials are the testosterone formulations used (testosterone gel vs i.m. testosterone undecanoate) and higher testosterone levels reached in the T4DM trial compared with the TRAVERSE trial. Incidence rates might be higher in clinical practice, as both trials excluded participants with baseline hematocrit exceeding 50%, thereby decreasing the likelihood of them developing erythrocytosis exceeding hematocrit thresholds. Additionally, the TRAVERSE trial also downtitrated testosterone to the lowest possible dosage whenever hematocrit exceeded 54%, whereas in clinical practice a dose reduction might be curbed or reverted due to patient preference or recurrence of hypogonadal symptoms.

Erythropoiesis takes place in the bone marrow and is regulated by EPO. EPO is a glycoprotein hormone that is primarily synthesized in the kidney and, to a smaller extent, in the liver. Specialized kidney cells sense pO_2_ and its decrease forms a strong signal to increase EPO production (25). These renal EPO-producing and oxygen-sensing (REPOS) cells are strategically located along the corticomedullary oxygen gradient in the juxtamedullary cortex, rendering them exquisitely sensitive to changes in pO_2_ (26). The potentially large amplitude of this effect can be seen in severe hypoxia, in which EPO production can increase up to a 1000-fold from baseline (27). A group of transcription factors named hypoxia-inducible factors (HIFs) play a central role in the molecular machinery that senses pO_2_ and translates this to EPO production in order to maintain red cell mass and thereby O_2_ delivery to the body (28). Briefly, HIFs are heterodimeric transcription factors consisting of an α- and β-subunit. There is a single β-subunit that is constitutively expressed and there are three known α-isoforms* –* all of which are O_2_-regulated: HIF-1α, HIF-2α, and HIF-3α. Several lines of evidence indicate that HIF-2α is the main regulator of EPO production (28). The α-subunits are principally regulated by two types of enzymes that collaboratively control their levels. Under normoxic conditions, the α-subunits are hydroxylated on two prolyl residues by the iron- and oxoglutarate-dependent HIF prolyl hydroxylases (prolyl hydroxylase domain (PHD) 1 to 3) (29). After hydroxylation, the von Hippel–Lindau (VHL) complex, consisting of the VHL tumor suppressor protein and several other proteins, is able to interact with HIF-α (30). The complex functions as a ubiquitin ligase that targets prolyl-hydroxylated HIF-α for proteasomal degradation. Under hypoxic conditions, the PHDs cease to hydroxylate HIF-α as they require O_2_ as a co-substrate. Consequently, the VHL complex is unable to interact with and ubiquitylate HIF-α, and thus its levels accumulate. It is important to realize that hypoxia is a relative term since pO_2_ varies widely between tissues and also within a tissue depending on the proximity of a cell to its supplying vasculature and arterial (oxygen-rich) end (31). Any decrease in pO_2_ that causes a biological effect in a cell, such as increased HIF-α stability, can arguably be called hypoxia (32).

While significant progress has been made, our understanding of the mechanism of action behind the stimulation of erythropoiesis by testosterone remains incomplete. Based on animal and human experiments, several mechanisms have been proposed, including increased EPO production, stimulation of colony-forming unit-erythroids (CFU-E: erythroid progenitors which differentiate into proerythroblasts), and increased incorporation of iron into erythrocytes (33). However, stimulation of EPO production as a mechanism was called into question by a secondary analysis of a testosterone dose–response study published in 2008 (17). While a dose-dependent increase in hemoglobin and hematocrit was observed after 20 weeks of testosterone administration, no associated increase in EPO was found. Blood samples from this same dose–response study were later used for measurement of the master iron regulator protein hepcidin (34). Hepcidin inhibits gut iron absorption, and thus its suppression increases iron availability (35). A potent dose-dependent suppression of serum hepcidin was found, and this suppression was stronger in older than younger men. It might therefore be speculated that increased iron availability as a result of testosterone-induced hepcidin suppression drives an increase in erythropoiesis. However, animal data show that hepcidin suppression is not required for the stimulation of erythropoiesis by testosterone (36). A six-month randomized-controlled trial provided evidence for a new EPO/hemoglobin ‘set point’ as a result of testosterone administration (37). EPO levels initially rise after which they slowly decrease back to baseline, but at a higher hematocrit. Thus, the EPO/hematocrit relationship curve is shifted to the right. Or, in other words, a new ‘set point’ is established at which the same EPO level is maintained, rather than suppressed, at a higher hematocrit. While research in mice shows that the increase in EPO production depends on DNA binding of the androgen receptor after ligand binding (38), it is unclear whether this is a direct effect mediated by binding to a promoter or enhancer region of the EPO gene or involves an intermediate step. Research on the effect of TTh on upstream regulators of EPO, such as the HIFs, VHL complex, and PHDs, is, unfortunately, lacking.

## Testosterone-induced erythrocytosis and thrombotic risk

Increased blood viscosity is associated with an increased risk of thrombosis (39), and hematocrit is an important determinant of blood viscosity, scaling exponentially in large and linearly in small vessels (capillaries) (40, 41). Thrombosis is also a major cause of morbidity and mortality in myeloproliferative disorders, such as PV, in which hematocrit is an important clinical finding and diagnostic criterion. However, hematocrit *per se* has been challenged as an important determinant of thrombotic risk in erythrocytosis (13). Several other factors involved in the pathophysiology of myeloproliferative disorders are thought to be the more likely culprit, although this does not preclude the possibility that increased hematocrit might compound risk in some of these conditions.

Several studies have looked at the association between erythrocytosis and thrombotic risk in the general population. The Tromsø Study, a prospective cohort study conducted in Tromsø, Norway, investigated the association between hematocrit and venous thromboembolism (VTE) (42). Using a multivariable model adjusted for age, smoking, and BMI, a significant positive association of hematocrit with VTE risk was found. A 5% hematocrit increase resulted in a hazard ratio of 1.33 (95% CI: 1.05–1.70) for total VTE and 1.66 (95% CI: 1.15–2.41) for unprovoked VTE in men, but no significant association was found in women (HR 1.11, 95% CI: 0.88–1.40 and HR 1.16, 95% CI: 0.81–1.67, respectively). The nonsignificant association in women might be related to the overall lower hematocrit levels in women. Caution should be taken when interpreting these results, as the possible inclusion of subjects with myeloproliferative neoplastic disorders might have also affected outcomes. A Danish prospective cohort study of the general population (the Copenhagen General Population Study) evaluated the association between hematocrit and both arterial and venous thrombosis (43). Subjects diagnosed with myeloproliferative neoplastic disorders were excluded from sensitivity analyses. Hematocrit ranges were categorized into the 0–5th, 6th–24th, 25th–75th (the reference), 76th–94th, and 95th–100th percentiles. No significant association was found in the highest percentile group with VTE (HR 1.26, 95% CI: 0.92–1.72) and ischemic stroke (HR 1.27, 95% CI: 0.91–1.75), but an increased risk was found for myocardial infarction (HR 1.46, 95% CI: 1.06–2.00). After multivariable adjustment for age, sex, smoking, alcohol consumption, body mass index, the Charlson comorbidity index, and antiplatelet therapy, the HRs were 1.34, 1.42, and 1.58 for VTE, ischemic stroke, and myocardial infarction, respectively, with again only the latter being statistically significant. The discrepancy in VTE between the Tromsø Study and Copenhagen General Population Study might be explained by the different approach in statistical analysis. Regardless, the HRs are similar and underscore that the risk of thrombosis due to erythrocytosis *per se* must be relatively modest compared with that seen in myeloproliferative neoplastic disorders.

While findings derived from the general population give some sense of the thrombotic risk associated with erythrocytosis, ultimately we are interested in erythrocytosis induced by TTh. The TRAVERSE trial found a nonsignificant higher incidence of VTE events in the testosterone-treated group compared with placebo (HR 1.46, 95% CI 0.92–2.32) (23). However, little can be concluded from this trial in regard to a potential link between TTh-induced erythrocytosis and thrombotic risk: the trial was not adequately powered for this endpoint, subjects with hematocrit >50% at baseline were excluded from enrollment, and in those who developed and maintained a hematocrit >54% after testosterone dose reduction, TTh was stopped. An individual participant dataset meta-analysis of testosterone trials comprising 3431 participants reports no increase in thrombotic events (44). Most of the analyzed studies, however, did not report these events and were not included in this analysis, rendering the meta-analysis strongly underpowered for an endpoint that has a relatively low background risk like thrombotic events. Importantly, randomized-controlled trials, such as the TRAVERSE trial, strictly adhere to clinical practice guidelines which recommend excluding participants with high hematocrit at baseline, and adjusting the dose or cease TTh upon developing erythrocytosis. Consequently, the participants that are presumably at highest risk of thrombosis as a result of increased hematocrit are excluded from these trials.

The best available evidence evaluating the link between TTh–induced erythrocytosis and thrombosis to date is therefore is a retrospective cohort study (45). A US multi-institutional database of 74 million patients was used to identify two cohorts of hypogonadal men who either did or did not develop erythrocytosis (defined as hematocrit ≥52%) on TTh. Propensity-score matching was used to address potentially confounding variables. The men who developed erythrocytosis had a higher risk of major adverse cardiac events (MACE) and VTE than those who did not (OR 1.35, 95% CI: 1.13–1.61). Interestingly, when the cutoff for erythrocytosis was set at a hematocrit of 50%, no significant difference between groups was observed.

Taken together, elevated hematocrit seems associated with an increased risk of both arterial and venous thrombosis, although high-quality evidence is lacking. Emphasis should also be placed on the word ‘associated’ as these studies are unable to infer causality. There might be one or more underlying factors that increase the risk of developing erythrocytosis and thrombosis on TTh. Moreover, there might be patient subgroups that are at increased risk, such as patients with type 2 diabetes mellitus (46). The increased risk, however, is likely modest in comparison with myeloproliferative neoplastic disorders such as PV. It is hard to estimate the risk of thrombosis in PV sans treatment, as treatment is promptly initiated after diagnosis. Nevertheless, 3 months after diagnosis, patients with PV have a 2.7- and 13.1-fold higher risk of arterial and venous thrombosis, respectively, compared with matched controls (47).

## Phlebotomy: a double-edged sword?

Several endocrine organizations mention therapeutic phlebotomy as a means of treating TTh–induced erythrocytosis, commonly with a hematocrit cutoff around 54%, as an alternative or adjuvant to testosterone dosage reduction (Table 1). In therapeutic phlebotomy, one unit of blood (500 mL) is collected at regular intervals. The goal is to decrease hematocrit to acceptable levels (e.g. <50%) and maintain these between phlebotomies. Periodic phlebotomy to persistently decrease hematocrit (even within the physiological range) has two consequences that we will discuss: a decrease in tissue pO_2_ and depletion of iron stores. An argument can be made that these biological changes that occur in concert might increase, rather than decrease, thrombotic risk, and therefore might offset* –* or even override* –* the potential benefit of correcting hematocrit.

It is important to emphasize that there is a vast difference between tissue pO_2_ and the arterial pO_2_ measurements that are routinely done with pulse oximetry. The pO_2_ in tissue depends on the volume of blood supplied per unit of time (perfusion), oxygen availability (hemoglobin concentration and saturation), and concurrent oxygen consumption. The mean pO_2_ varies widely between tissues, with the majority of tissue pO_2_ ranging between 23 and 70 mmHg (48). Within tissues, the pO_2_ also varies significantly. As O_2_ is continuously consumed, there will be a steep pO_2_ gradient radially and longitudinally along the blood capillaries, with the highest value at the arterial and the lowest at the venous end (31, 32). Assuming unchanged plasma volume, a decrease in hematocrit implies there are fewer red blood cells in the capillary and thus less O_2_-bound hemoglobin from which the O_2_ can dissociate and consequently a decreased tissue pO_2_. However, a decrease in hematocrit also increases blood flow as a result of decreased blood viscosity (40), thus increasing the rate of oxygen supply. While not researched in humans, animal data show that the net result of these two competing mechanisms on tissue pO_2_ follows the direction of hematocrit change when hematocrit is in the range of 20 to 60% (49). In other words, within this range, any increase in hematocrit increases tissue pO_2_ and vice versa.

Changes in tissue pO_2_ are relevant as they activate oxygen-sensing pathways. The K_M_ for oxygen of the earlier mentioned PHDs far exceeds tissue O_2_ concentrations. This enables PHD activity to be modulated over the entire physiological range of intracellular pO_2_ (50), implying that any change in hematocrit, and therefore tissue pO_2_, affects HIF stability.

Why would an increase in HIFs be relevant? Persistent elevations of HIFs in platelets, vascular endothelial cells, blood cells, and other cells might increase thrombotic risk (14). For example, Chuvash erythrocytosis is a congenital disorder caused by a missense mutation in the *VHL* gene. This impairs the rate at which the VHL complex ubiquitylates HIF-α (51). HIF-α therefore accumulates, which leads to increased expression of downstream target genes, including *EPO*, which is responsible for the erythrocytosis observed in this condition, but also various proteins of the coagulation cascade and fibrinolysis pathways (14). Notably, phlebotomy increases the risk of thrombosis in Chuvash erythrocytosis, likely because it further stimulates HIF-dependent pathways that affect thrombotic risk independently of hematocrit (52, 53), possibly through the upregulation of tissue factor, thrombospondin, and other factors by HIF (13). The routine use of phlebotomy as a treatment is therefore specifically not advised in Chuvash erythrocytosis (54). Hypoxia-inducible factor prolyl hydroxylase inhibitors (HIF-PHIs) that inhibit PHD activity, and thereby prevent HIF-α degradation, also increase thrombotic risk (55).

Would correcting hematocrit by periodic phlebotomy not return HIF levels to their state prior to initiating TTh and thus be harmless? The answer to this question hinges on the mechanism through which TTh induces erythrocytosis. As mentioned earlier, testosterone stimulates erythropoiesis through an initial rise in EPO, establishment of a new EPO/hemoglobin set point, and a parallel decrease in hepcidin. An increase in HIF, in particular HIF-2α, increases EPO production (28), which would establish a new EPO/hemoglobin set point and could be the underlying mechanism for testosterone’s recalibration of the EPO/hemoglobin set point. Additionally, increased HIF-α activity suppresses hepcidin (56), which could thus also potentially explain testosterone’s hepcidin-suppressive effect. If this is indeed the underlying mechanism of action, then the TTh-induced erythrocytosis returns HIF levels to baseline, whereas decreasing hematocrit to within the reference range by periodic phlebotomy would further exacerbate HIF upregulation* –* similar to the situation in Chuvash erythrocytosis, albeit of a different magnitude. This further highlights the need for research in delineating the mechanism of action of TTh-induced erythrocytosis. EPO levels will likely also remain elevated, as correction of hematocrit by phlebotomy will maintain hematocrit below the new EPO/hemoglobin set point. It is noteworthy that the use of EPO mimetics and recombinant EPO is also associated with increased thrombotic risk (57).

The question that remains is whether decreased tissue pO_2_ in response to therapeutic phlebotomy in TTh-induced erythrocytosis increases HIF activity sufficiently to also confer a thrombotic risk that might offset or override the potential benefit of correcting increased hematocrit. In contrast to the state prior to initiating TTh, iron depletion might further compound the effect on HIFs since the PHDs require iron to catalyze prolyl hydroxylation (58). PHD activity thus decreases in an iron-depleted state, and HIF-α subunits will accumulate and heterodimerize with the constitutively expressed β-subunit to regulate gene expression. Some clues might be derived from iron deficiency anemia, which is associated with increased thrombotic risk (59), although, obviously, great caution should be taken when extrapolating these results to a phlebotomy-induced ‘relative anemia.’

**Figure 1:**
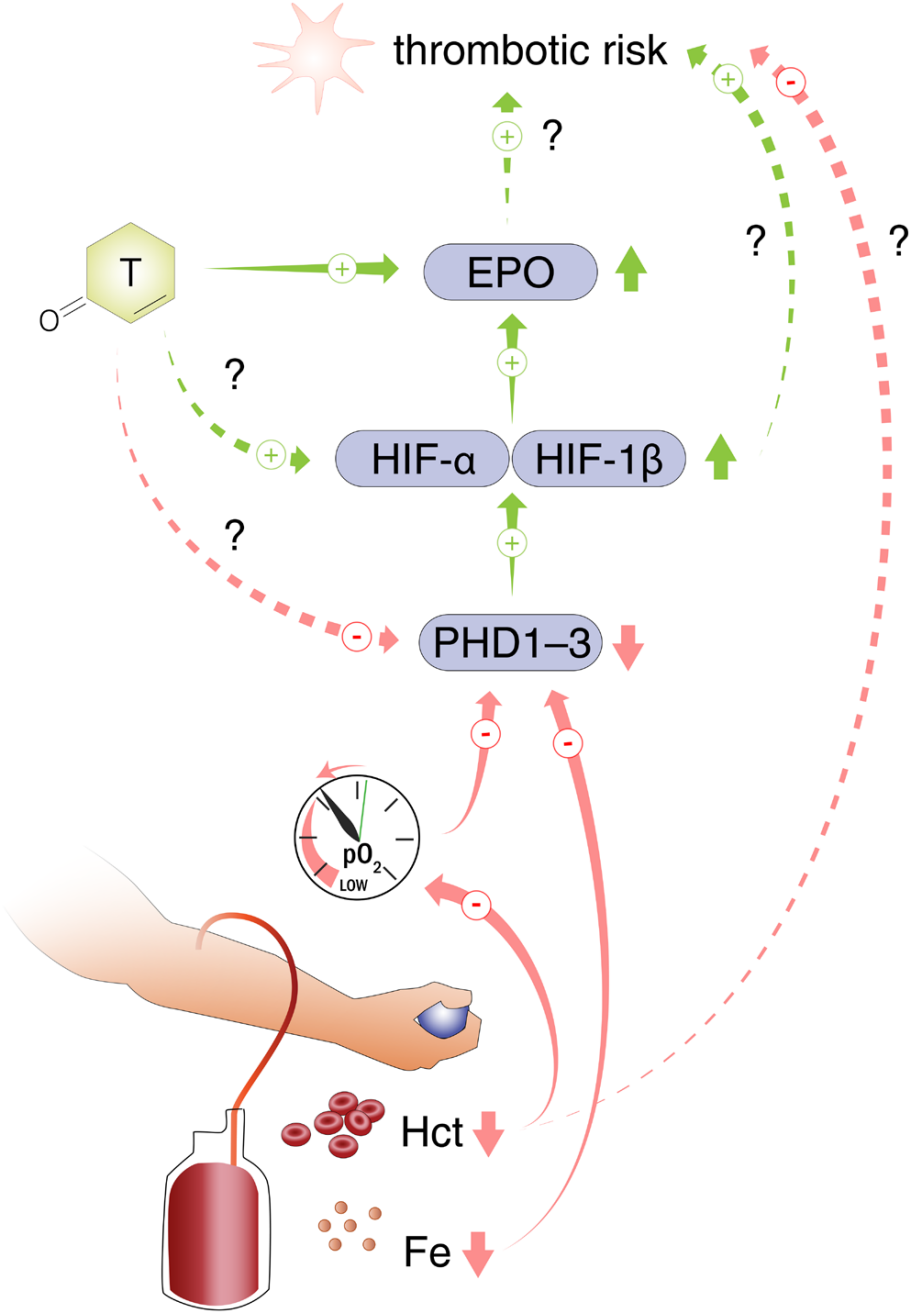
Testosterone therapy (TTh) can cause erythrocytosis in some patients. Testosterone stimulates erythropoiesis through an initial rise in erythropoietin (EPO), the establishment of a new EPO/hemoglobin ‘set point’, and a parallel decrease in the master iron regulator protein hepcidin, as well as several other potential mechanisms (not illustrated). Therapeutic phlebotomy might be initiated to decrease hematocrit to acceptable levels and maintain these between phlebotomies. We hypothesize that this might impose a risk by decreasing tissue pO_2_ and depleting iron stores, which jointly inhibit PHD1-3 activity leading to HIF-α stabilization. Persistent elevations of HIFs might increase thrombotic risk (possibly partly through increased EPO), which could offset or override, the potential benefit of correcting hematocrit. The underlying mechanism of T’s stimulation of erythropoiesis is important: if this functions through stabilization of HIF-α* –* whether or not through suppression of PHD activity* –* therapeutic phlebotomy might further exacerbate HIF upregulation. Abbreviations: T, testosterone; EPO, erythropoietin; HIF, hypoxia-inducible factor; PHD, prolyl hydroxylase domain; pO_2_, partial pressure of oxygen; Hct, hematocrit; Fe, iron.

In summary, the practice of therapeutic phlebotomy for treating TTh-induced erythrocytosis might potentially increase, rather than decrease, thrombotic risk* –* through the HIF pathway* –* by the combined action of depleting iron stores and decreasing tissue pO_2_ (see Fig. 1).

## Conclusion

TTh stimulates erythropoiesis, which can lead to erythrocytosis in some patients. The TTh-induced increase in hematocrit might parallel an increase in thrombotic risk, although the amplitude of this risk is most likely lower than seen in erythrocytosis caused by myeloproliferative disorders. Therapeutic phlebotomy can decrease hematocrit to within the reference range, which purportedly negates this risk. Evidence for this purported negated risk predominantly stems from its efficacy in lowering thrombotic risk in erythrocytosis from causes other than TTh, such as PV. A consequence of periodic phlebotomy is lowering tissue pO_2_ and, eventually, depletion of iron stores. Inevitably, biological hypoxia-sensing and iron-dependent pathways are activated, which are linked to an increased thrombotic risk. The question that remains is to what side the proverbial coin falls when applying therapeutic phlebotomy to TTh-induced erythrocytosis: an increased or decreased thrombotic risk? In the absence of evidence for its efficacy, lowering testosterone dosage, as well as targeting risk factors for developing erythrocytosis, such as smoking and severe obstructive sleep apnea, should be explored first. Only after this should, in shared decision-making* –* highlighting the uncertainties of its efficacy and safety* –* be decided upon the application of therapeutic phlebotomy. Future trials should evaluate how TTh affects the HIF-EPO pathway and how its resulting erythrocytosis might impact thrombotic risk. Evidence should also be gathered regarding the efficacy and safety of phlebotomy as a potential remedy for TTh-induced erythrocytosis.

## Declaration of interest

The authors declare that there is no conflict of interest that could be perceived as prejudicing the impartiality of this work.

## Funding

This work did not receive any specific grant from any funding agency in the public, commercial, or not-for-profit sector.

## Author contribution statement

PB – writing of the manuscript. TV – review of the manuscript. DLS – review of the manuscript.
